# The Emerging Role of Biomarkers in the Diagnosis of Gestational Diabetes Mellitus

**DOI:** 10.3390/jcm7060120

**Published:** 2018-05-23

**Authors:** Natassia Rodrigo, Sarah J. Glastras

**Affiliations:** 1 Department of Diabetes, Endocrinology & Metabolism, Royal North Shore Hospital, St Leonards, Sydney 2065, Australia; natassia.rodrigo@sydney.edu.au; 2 The Kolling Institute of Medical Research, St Leonards, Sydney 2065, Australia; 3 Faculty of Medicine, The University of Sydney, Sydney 2006, Australia

**Keywords:** gestational diabetes mellitus, biomarkers, pathophysiology, predictive diagnosis

## Abstract

Gestational diabetes mellitus (GDM) is a common complication of pregnancy; its rising incidence is a result of increased maternal obesity and older maternal age together with altered diagnostic criteria identifying a greater proportion of pregnant women with GDM. Its consequences are far-reaching, associated with poorer maternal and neonatal outcomes compared to non-GDM pregnancies, and GDM has implications for metabolic health in both mother and offspring. Objective markers to identify women at high risk for the development of GDM are useful to target therapy and potentially prevent its development. Established clinical risk factors for GDM include overweight/obesity, age, ethnicity, and family history of diabetes, though they lack specificity for its development. The addition of biomarkers to predictive models of GDM may improve the ability to identify women at risk of GDM prior to its development. These biomarkers reflect the pathophysiologic mechanisms of GDM involving insulin resistance, chronic inflammation, and altered placental function. In addition, the role of epigenetic changes in GDM pathogenesis highlights the complex interplay between genetic and environmental factors, potentially offering further refinement of the prediction of GDM risk. In this review, we will discuss the clinical challenges associated with the diagnosis of GDM and its current pathophysiologic basis, giving rise to potential biomarkers that may aid in its identification. While not yet validated for clinical use, we explore the possible clinical role of biomarkers in the future. We also explore novel diagnostic tools, including high throughput methodologies, that may have potential future application in the identification of women with GDM.

## 1. Introduction

Gestational diabetes mellitus (GDM), defined as glucose intolerance or hyperglycaemia that first manifests and is diagnosed during gestation, affects up to one in ten pregnancies worldwide [1]. Pregnancy can induce significant insulin resistance in women with pre-disposing factors and, coupled with an insufficient compensatory insulin secretory response, maternal hyperglycaemia ensues. Women with GDM have increased rates of assisted delivery and caesarean section, and neonatal complications including birth injury, respiratory distress syndrome, and hypoglycaemia [2]. Moreover, poorly controlled GDM or hyperglycaemia in pregnancy contributes towards foetal hyperinsulinaemia and relative foetal hyperglycaemia. In turn, this leads to foetal overgrowth, including macrosomia (birth weight over 4 kg) or large for gestational age (LGA; birth weight above the 90th centile for gestational age and gender) neonates.

Women with GDM are more likely to experience recurrent GDM in future pregnancies, and they have an increased risk of developing type 2 diabetes (T2D) and cardiovascular disease in adulthood [3,4]. In addition, intrauterine exposure to the maternal milieu associated with GDM can result in negative foetal programming in the offspring, leading to an increased risk of metabolic conditions such as obesity and T2D in adult life, independent of genetic and postnatal environmental factors [5,6,7,8] (Table 1). These potential adverse outcomes in both mother and offspring underpin the importance of correctly diagnosing and managing GDM.

Strategies to identify women at risk of GDM as early as possible in pregnancy may be advantageous, as intrauterine exposure to hyperglycaemia may be circumvented. Clinical risk factors for GDM are well described [9]. However, previous studies in which intensive lifestyle advice has been given to women with strong risk factors for GDM development have not proven beneficial in preventing its development [10]. Identification of biomarkers, readily obtained from blood samples in early pregnancy, may complement existing clinical risk factors to identify women at high risk of developing GDM. Furthermore, such biomarkers could distinguish women who may benefit from targeted strategies to reduce GDM development. In the present review, we will discuss the current methodologies used in the diagnosis of GDM, as well as recent advances in defining the role of predictive biomarkers for GDM.

## 2. Current Diagnostic Tools for the Diagnosis of GDM

The Hyperglycaemia and Adverse Pregnancy Outcome (HAPO) study represents the landmark trial in GDM, clearly establishing the strong association of maternal glucose levels with adverse pregnancy outcomes [11]. Further, the demonstrable reduction in maternal and foetal adverse events after lowering maternal glucose has led to international guidelines, including endorsement by the International Association of Diabetes and Pregnancy Study Groups (IADPSG), advocating that screening for GDM should occur at 24–28 weeks gestation in all pregnant women [12,13,14,15,16]. The impact of the revised IADPSG diagnostic criteria, with lower diagnostic glucose thresholds, has further exacerbated the growing problem of GDM [17]. The reported prevalence of GDM using IADPSG criteria varies from 3.5% to 45.3%, with the application of the revised IADPSG criteria universally increasing prevalence overall, with the absolute increase by as much as 33% [18,19,20,21,22,23].

Women with pre-gestational type 1 and type 2 diabetes have evidently poorer pregnancy outcomes than the general population, including a significantly higher risk of congenital abnormalities and a 4-fold higher risk of perinatal mortality [24,25,26,27]. As there is an increasing prevalence of undiagnosed diabetes, particularly T2D, prior to pregnancy, diagnosis of pre-existing diabetes in early pregnancy is clearly of clinical benefit [28]. Existing international guidelines recommend testing for pre-gestational diabetes in early pregnancy in high-risk women, identified by BMI > 30 kg/m^2^, history of GDM or impaired glucose tolerance [15,16,29]. The methodology utilised to identify women with pre-gestational diabetes, however, differs between guidelines [15,17,29,30].

Separate to establishing pre-gestational diabetes, there is currently no evidence of benefit to mother or neonate to substantiate the extrapolation of diagnostic criteria for GDM, based on a 75 g OGTT at 24–28 weeks, to earlier gestation [31]. Furthermore, performing an OGTT in early pregnancy has several disadvantages. It requires fasting overnight, 3 sets of blood tests temporally spaced over 2 h, and it is often complicated by nausea and vomiting secondary to delayed gastric emptying, especially in early pregnancy [32]. An alternate approach to diagnose diabetes in early pregnancy is to measure glycosylated haemoglobin (HbA1c). HbA1c has less inter-laboratory variation compared to plasma glucose levels collected during OGTT, less intra-individual variability, and it is not affected by diurnal variation, meals, fasting, acute stress, or drugs that influence glucose metabolism [33]. While various studies have explored the utility of HbA1c as an alternate diagnostic tool for GDM, there are weaknesses associated with HbA1c unique to pregnancy. An understanding of ‘normal’ ranges of HbA1c in pregnancy is not fully elucidated; O’Conner et al. suggested trimester specific reference intervals for HbA1c, in the first trimester 4.8–5.5% (29–37 mmol/mol), second trimester 4.4–5.4% (25–36 mmol/mol), and third trimester, 4.4–5.4% (25–36 mmol/mol) [34]. However, HbA1c is inversely linked to plasma volume changes and accelerated red blood cell turnover, and thus is inherently variable in the pregnant state and vulnerable to artificial cut-offs. A recent retrospective Australian cohort study demonstrated that a baseline HbA1c > 5.9% was correlated with an increased risk of LGA neonates, macrosomia, caesarean section, and hypertensive disorders [35]. Similarly, an HbA1c of 5.4% has been demonstrated to have 95% specificity, 27% specificity, and a negative predictive value of 91% for GDM at 24–28 weeks of gestation [32]. The lack of universal validity of early pregnancy OGTT and HbA1c remains problematic and demonstrates a weakness in current methodologies used to diagnose GDM. Greater accuracy could be improved through the incorporation of biological markers, so called ‘biomarkers’, into diagnostic algorithms. Effective delivery of finite antenatal resources necessitates better understanding of the natural history and pathophysiology of GDM, ideally aimed to identify at-risk women early in pregnancy and prevent GDM development by strategic intervention.

## 3. Clinical Predictors of Foetal Overgrowth

The most common and preventable consequence of undiagnosed and untreated GDM is foetal overgrowth, manifesting as LGA or macrosomia. Asymmetric overgrowth may occur, in which the abdominal circumference is disproportionally high, often accompanied by increased rates of shoulder dystocia, complicating normal vaginal delivery [36]. Clinical risk factors for LGA and macrosomia include previous macrosomia, multiparity, delivery beyond 40 weeks gestation, obesity, and diabetes [37,38]. The role of maternal hyperglycaemia in foetal overgrowth syndromes is firmly established. Normalisation of maternal blood glucose levels, as achieved in women with well managed diet-controlled GDM, reduces rates of foetal overgrowth, comparable to the non-GDM population [39]. Indeed, a greater foetal abdominal circumference measured as early as 16 weeks gestation, is associated with macrosomia and LGA, in women with maternal glucose intolerance [40]. Nonetheless, factors separate to maternal glucose levels should not be ignored. Our research examining the pregnancy outcomes of women with type 1 diabetes highlights that, even when excellent glycaemic control is achieved, foetal overgrowth can still ensue, suggesting factors separate to maternal hyperglycaemia are at play [41].

The role of maternal body weight is increasingly appreciated to impact foetal growth. Higher maternal BMI in early pregnancy is associated with macrosomia [42,43]. In addition, increased gestational weight gain above the Institute of Medicine guidelines is associated with LGA [44]. The long-term consequences of maternal obesity on the risk of developing multiple features of the metabolic syndrome and T2D in both mother and offspring further emphasises the importance of optimising maternal weight for optimal maternal-foetal health [5,45,46,47,48,49]. Preconception optimisation of body weight and standard lifestyle advice in pregnancy with respect to healthy eating and activity are the key strategies to prevent foetal overgrowth. An inadvertent consequence of GDM treatment is reduced gestational weight gain which may have benefit beyond glucose lowering [50,51]. Focusing attention away from a glucose-centric approach to a more holistic understanding of the metabolic perturbations in pregnancy is likely to lead to better methods to predict neonates at risk of foetal overgrowth and its consequences. 

## 4. Clinical Predictors of GDM

The trajectory of GDM incidence across the world is increasing at an alarming rate, now estimated to affect between 5% and 10% of pregnancies, compared to 3–5% of pregnancies prior to the 21st century [52]. This is due to the increase in incidence of clinical predictors of GDM, which overlap considerably with clinical predictors of foetal overgrowth. In particular, the increased prevalence of overweight and obesity, affecting up to 50% of women of reproductive age has increased the incidence of GDM globally [53,54]. Further, the advancing maternal age of the reproductive population has also contributed to the increased prevalence of maternal adverse outcomes. Fushs et al. found higher rates of chronic hypertension, assisted reproduction techniques, pre-gestational diabetes, GDM, assisted delivery, and preterm birth in women older than 35 years [55]. GDM risk in women greater than 30 years of age is up to four-fold higher than women younger than 30 years [56]. Women with polycystic ovary syndrome (PCOS) have an increased risk of GDM in pregnancy [57]. Importantly, PCOS is a clinical diagnosis with pathogenesis underpinned by insulin resistance, resulting in an anovulatory menstrual cycle and associated hyperandrogenism. Furthermore, clinical risk factors include a previous history of GDM, a family history of T2D or ethnicity including Asian, Middle Eastern, non-white African, Hispanic, and indigenous populations such as the Australian Aboriginals (Box 1). With the rising epidemic of T2D globally and the trend towards multicultural societies, these clinical risk factors are increasingly affecting a large proportion of pregnant women.

Box 1 **Clinical risk factors for gestational diabetes mellitus.**

Previous hyperglycaemia in pregnancy

Previously elevated blood glucose level

Maternal age ≥ 40 years

Ethnicity: Asian, Middle Eastern, non-white African, Hispanic, Aboriginal, Torres Strait Islander, Pacific Islander, Maori, American Indian

Family history DM (1st degree relative with diabetes or a sister with hyperglycaemia in pregnancy)

Pre-pregnancy BMI > 30 kg/m^2^

Previous macrosomia (baby with birth weight >4500 g or >90th centile)

Polycystic ovarian syndrome

Medications: corticosteroids, antipsychotics

These clinical risk factors are endorsed by leading organisations affiliated with diabetes in pregnancy, including the International Association of Diabetes and Pregnancy Study Groups (IADPSG) [17].

## 5. Pathophysiology of GDM

Pregnancy is a state of metabolic flux, in which the maternal system adapts to facilitate the presence of the maternal foetal unit. In pregnancies unaffected by GDM, maternal tissues gradually develop insulin insensitivity, with a reciprocal increase in insulin secretion by 200% and reduced whole body glucose disposal by 50% to maintain euglycaemia [58]. In GDM, there exists a relative deficiency of insulin to overcome this increased resistance, such that hyperglycaemia develops [58]. Particular ethnic groups, such as South-East Asian populations, are known to have fewer insulin secretory cells, namely pancreatic beta cells, which may at least partly explain the increased incidence of GDM in these populations [59].

Adiponectin, in its role as a modulator of glucose metabolism, has an inverse relationship to insulin resistance, with low adiponectin levels observed prior to the development of T2D [60]. Adiponectin increases fatty acid oxidation and inhibits hepatic glucose production [61]. Adiponectin is also produced by the placenta during pregnancy, with significant downregulation of placental adiponectin mRNA levels demonstrated in GDM placenta compared to normal placenta [62]. Concomitant elevations in adiponectin receptor 1 mRNA expression in GDM placentae suggest that metabolic changes can influence adiponectin receptor expression [62]. Low adiponectin levels in the third trimester are associated with the presence of GDM, independently of maternal weight [63]. Furthermore, Lain et al. demonstrated a significant reduction in adiponectin levels from as early as 9 weeks gestation in women who develop GDM [64]. Interestingly, adiponectin levels are reduced by pro-inflammatory cytokines [61], highlighting the interaction between inflammation and metabolic dysregulation.

Human placental lactogen (hPL), the major hormone produced by the placenta, increases exponentially during pregnancy. It acts to endorse maternal beta cell expansion and insulin production by working in concert with prolactin to increase phosphorylation of intracellular signalling pathways and pancreatic islets specific transcription factors [65,66]. Human placental growth hormone (hPGH) concentrations also increase during pregnancy, increasing peripheral insulin resistance [65,67]. Further, intracellular signalling changes in skeletal muscle and adipose tissue lead to inhibited signalling, reduced glucose transporter translocation and thereby decreased glucose uptake [58,68,69]. Further understanding of the cellular mechanisms that occur during pregnancy and their dysfunction in GDM may direct meaningful searches for biomarkers that have prospective clinical utility.

## 6. Biomarkers as Predictive Tools to Identify GDM

Biological markers, or ‘biomarkers’, consist of any substance in the body that can be quantified and assessed to represent normal physiology, a pathogenic pathway, or pharmacological response to a therapeutic intervention [70]. Biomarkers are classified as either antecedent markers that assess the risk of developing a disease, or as screening tools to identify disease in the subclinical phase designed to diagnose, prevent progression or predict response to therapy [71]. Many biomarkers have been investigated in the field of GDM research (Table 2), revealing a greater understanding of the complexities of GDM pathophysiology, as well as serving as potential diagnostic markers.

### 6.1. Metabolic Biomarkers and GDM

Underpinned by the known dysregulation of glucose metabolism in GDM, metabolic predictors have been investigated as predictive tools to identify women at risk of GDM. Higher insulin resistance indices in the first trimester of pregnancy, as determined by the homeostasis model assessment (HOMA) utilising fasting serum glucose and insulin measures, are associated with increased risk of GDM [72,73]. Grewal et al. found that Asian Indian women with higher measures of insulin resistance in the first trimester of pregnancy were at higher risk of GDM at 24–28 weeks gestation [73]. In addition, fasting insulin levels in early pregnancy predict GDM at 24–28 weeks gestation [74]. Insulin resistance alone is insufficient as a predictive marker as not all studies conclusively demonstrate this link [75].

The physiology of pregnancy is associated with a progressive decline in insulin sensitivity and a concomitant increase in insulin resistance, with this inverse relationship peaking in effect in the third trimester and subsiding post-delivery. Measures of insulin sensitivity in the first trimester, using the Matsuda index (composite insulin sensitivity from OGTT), quantitative insulin sensitivity check index, and HOMA for sensitivity, have been investigated as possible predictors of GDM [73]. As the relationship between insulin sensitivity and GDM precedes its development, further validation of insulin sensitivity as a predictor of GDM would be useful, allowing opportunities for intervention prior to GDM development.

Sex hormone binding globulin (SHBG), a glycoprotein that binds androgen and estrogen, has an inverse relationship with elevated insulin levels in both the first and early second trimesters of pregnancy in women who later develop GDM [76,77]. Though initially showing promise as a predictive tool, the significance of this predictive potential was lost once BMI, ethnicity, and family history were taken into consideration, highlighting the importance of a biomarker to have additive predictive potential, beyond standard clinical risk factors.

Lipid metabolism is altered in pregnancy, with first and second trimesters of pregnancy signifying the greatest period of maternal body fat accumulation as a consequence of increased lipid synthesis [78,79]. Lipid levels increase gradually throughout pregnancy, peaking in late pregnancy [79,80]. Triglycerides do not directly cross the placenta, however lipoprotein receptors on the placenta allow fatty acid movement down the maternal-foetal gradient [79]. These changes are exaggerated in GDM, with higher triglyceride levels found in all trimesters [81,82]. Consolidating the importance of maternal triglycerides, circulating levels in the third trimester are positively associated with foetal birthweight independently of GDM [83,84,85]. Conversely, maternal high-density lipoprotein (HDL) levels are inversely associated with foetal macrosomia [85,86]. Though the association between perturbed maternal lipid levels and GDM are established, their role as predictive biomarkers remains unclear.

### 6.2. Inflammatory Biomarkers and GDM

Obesity is a known risk factor for the development of GDM [13]. It is a state of chronic low-grade inflammation, resulting from exposure to excess nutrients and energy [83]. This pro-inflammatory environment alters the metabolic cellular processes within adipose tissue, the liver, and pancreas, as manifest by altered levels of several adipokines, chemokines, and cytokines [83,84,85]. Tumour necrosis factor alpha (TNFα), produced by the placenta, has been implicated as a potential mediator of the insulin resistance of pregnancy. Syngelaki et al. found that maternal TNFα levels measured in serum at 11–13 weeks gestation was associated with subsequent GDM in a case-control study of 1000 women from the UK [87]. Another marker of inflammation, C-reactive protein (CRP) has also been implicated as a predictor of GDM [75,88]. Nonetheless, the relationship between CRP and GDM development was attenuated after adjustment for maternal BMI [88]. Moreover, CRP is a non-specific marker that can reflect systemic inflammation from a variety of causes and therefore lacks specificity with respect to GDM. It is unlikely to have clinical utility as a biomarker for the diagnosis of GDM.

Interleukin-6 (IL-6) is a circulating proinflammatory cytokine, 30% of which originates from adipocytes [89]. Circulating levels are higher in obese versus non-obese individuals, with direct correlation between IL-6 and measures of adiposity, including BMI and percent fat mass [90,91,92]. Chronic exposure to elevated IL-6 is associated with the development of insulin resistance [93]. Even in the absence of maternal obesity, maternal plasma levels of IL-6 are positively associated with GDM [94]. Longitudinal studies have demonstrated heterogeneity in the progression of these levels over the course of a normal pregnancy [95,107]. Studies are yet to establish if IL-6 levels prospectively predict the development of GDM, rather than simply being a byproduct of the pathophysiologic state of GDM.

### 6.3. Placental Biomarkers and GDM

The placenta itself is a source of inflammation; in obesity, the placenta is a source of even greater levels of inflammatory cytokines including IL-1, TNFα, and IL-6 [108]. Though there appears to be adaptive mechanisms in the placenta to limit foetal exposure to inflammation [109], altered glucose transport across the placenta through modulation of glucose transporters (GLUTs) has been demonstrated [96]. Specifically, GDM pregnancies are associated with increased placental GLUT9a expression, exacerbated by exposure to exogenous insulin [97]. The expression of basal membrane GLUT1 is stable at glucose concentrations in the physiological range, altering only in the extremes of glucose concentration [98]. Despite these adaptations, the placenta in GDM pregnancies exhibit a 2–3-fold increase in glucose uptake [110]. Such evidence distinguishes the placenta as a regulator of the foetal environment, with attempts to attenuate the impact of maternal metabolic dysregulation on foetal development occurring at a cellular level. Greater understanding of the physiology of the placenta may lead to the discovery of future biomarkers for GDM and its associated foetal complications.

## 7. Genetic and Epigenetic Biomarkers and GDM

Genome wide association studies (GWAS) have established up to 41 genetic risk loci for T2D, shedding light, at least in part, on the hereditability of diabetes [111,112]. Few GWAS studies have specifically examined GDM, instead relying on shared genetic signatures with T2D [113]. Common genetic variants, *CDKAL1* and *MTNR1B*, found in T2D are significantly associated with GDM [114]. Specifically, *CDKAL1* interacts with signalling molecules on pancreatic beta cells responsible for beta cell survival [115], and *MTNR1B* is expressed on beta cells and modulates insulin secretion [99]. Given the known pathogenic role of insufficient insulin secretion in GDM, these genetic variants hold promise as potential markers predicting GDM development though alone are likely to lack sensitivity.

Epigenetics, defined as chromosomal changes, not affecting the underlying DNA sequence, may have a role in GDM pathogenesis with potential utilisation of epigenetic signatures as predictive markers of GDM [100]. Most research related to epigenetic impact on the development of GDM has occurred using animal models, mostly achieved by altering maternal dietary factors, demonstrating increased epigenetic modifications in association with metabolic dysregulation after exposure to high fat diets [101,102,103]. A recent human study identified several genes (*COPS 8*, *PIK3R5*, *HAAO*, *CCDC124*, and *C5orf34*) with differential methylation in GDM compared to matched controls [104]. DNA methylation is the most commonly studied epigenetic modification, whereby the covalent modification of the fifth carbon of cytosine typically results in reduced gene transcription. A study by Xie et al. demonstrated a correlation between gestational glucose level and DNA methylation of the PPAR gamma coactivator 1 alpha (PGC1A) promotor [105]. The role of PGC1A methylation is known to align with common pathophysiological mechanisms associated with GDM, namely decreased insulin secretion in the pancreas, and increase hepatic glucose production in the liver [106]. Further, genome wide comparative methylome analysis of umbilical cord blood obtained from GDM-exposed neonates demonstrated epigenetic changes in areas known to be associated with type 1 diabetes, neuron development pathways, and immune pathways [116]. Such findings suggest that epigenetic modifications, particularly DNA methylation, play a role in the aetiology of GDM. Research is yet to establish if important epigenetic changes occur prior to GDM and whether they can predict its development.

## 8. Future Directions

**Figure 1:**
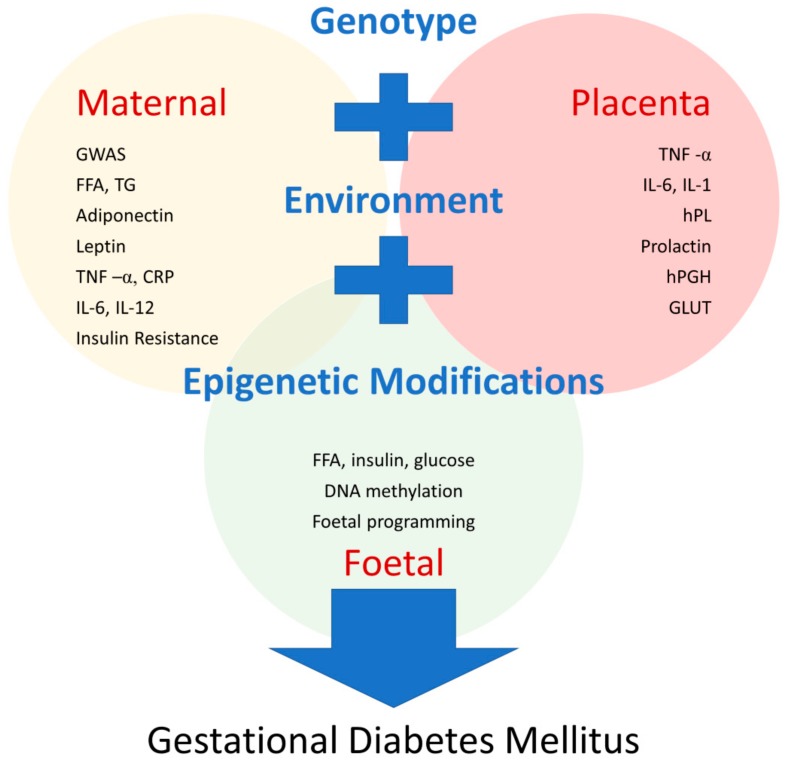
Known pathophysiologic mechanisms in GDM. These mechanisms give rise to potential biomarkers to predict GDM development. There is a complex interplay between maternal, foetal, and placental factors at a cellular level involving genetic, environmental, and epigenetic mechanisms. Abbreviations: GWAS, genome-wide association study; FFA, free fatty acids; TG, triglycerides; TNF-α, tumour necrosis factor alpha; CRP, C reactive protein; IL-6, interlukin-6; IL-12, interlukin-12; IL-1, interlukin-1; hPL, human placental lactogen; hPGH, human placental growth hormone; GLUT, glucose transporter.

Recent development of high throughput technologies has allowed greater exploration of the metabolome to a degree that was previously unfathomable. Metabolomic studies, whereby biological samples undergo qualitative and quantitative analysis, usually through mass spectrometry and nuclear magnetic resonance spectroscopy, to identify small molecule metabolic products, have already provided large amounts of information on the role of lipids, carbohydrates, and amino acid metabolites in T2D [117,118,119]. Similar insights are emerging in GDM, with metabolomic signatures demonstrating links between fatty acid metabolism and maternal glucose handling [120,121]. The complexities of the lipid metabolic pathways are illuminated through the application of mass spectrometry techniques; indeed, lipidomics is providing a fascinating insight into the mechanisms of metabolic diseases, raising the possibility of novel biomarkers for clinical application in GDM [122]. Using these novel technologies, the potential for unparalleled studies investigating the cellular processes that underpin GDM are likely to widen our understanding of its pathogenesis (Figure 1). Moreover, the application of machine deep learning and mathematical modelling to process massive datasets has the potential to lead to better predictive models of GDM from early gestation. An unresolved challenge is finding ways to process huge volumes of data in a time- and cost-effective manner.

While significant advances have been made in the field of biomarkers in GDM diagnosis, currently none have sufficient validity for clinical practice. Many potential biomarkers have been identified, though their significance in the pathophysiology of GDM requires further exploration. The heterogeneity of the GDM population adds further complexity in identifying a universal biomarker that has the sensitivity and specificity to adequately predict disease. Furthermore, well-designed prospective studies are required before the clinical use of biomarkers reaches prime time.

## 9. Conclusions

GDM affects a significant proportion of pregnant women and is likely to become even more prevalent as rates of obesity rise globally. Its development and complications could be circumvented if accurately predicted in early pregnancy and effective interventions initiated. Multiple clinical risk factors for GDM have been established, but are insufficient to accurately predict risk. Though several biomarkers for GDM have been investigated and contribute to our understanding of its pathogenesis, so far none have demonstrated adequate robustness to be added to clinical algorithms for GDM prediction. The integration of high throughput methodologies offers novel insights into the role of genetic variants, epigenetics, and metabolomics in the pathogenesis of GDM. The opportunity to apply predictive modelling in the subclinical phase of GDM is emerging as an exciting area for future research and development. These new technologies are inherently complex and discerning their role beyond the scope of research will be critical. Issues related to validity across populations, reproducibility, and selectivity need to be resolved prior to clinical implementation. So long as issues related to cost-effectiveness and universal access can be overcome, complex biomarkers are likely to prove invaluable in the diagnosis of GDM.

## Figures and Tables

**Table 1:** Maternal and foetal complications of GDM.

Maternal Complications	Foetal Complications
Short term:	Short term:
Hypertensive disorders in pregnancy	Large for gestational age/macrosomia
Failure to progress in labour	Perinatal death
Caesarean section	Shoulder dystocia and related birth injuries
Instrumental delivery	Neonatal hypoglycaemia
Pre-term delivery	Increased admission to NICU
Preeclampsia	Hyperbilirubinaemia
Long term:	Long term:
Recurrent GDM in subsequent pregnancies	Type 2 diabetes
Type 2 diabetes	GDM (females only)
Cardiovascular disease	Obesity

**Table 2:** Biomarkers under investigation in GDM.

Biomarker	Advantages	Disadvantages
Insulin Resistance	Elevations in first trimester are associated with higher risk of GDM prediction at 24–28 weeks in certain subpopulations of GDM women [71,72,73]	Not conclusively demonstrated as predictive in all studies [74]
Insulin sensitivity	Early studies demonstrate association with GDM [72]	Heterogeneity of calculation methods used in studies limits universal comparison [72]
SHBG	Inverse relationship with elevated insulin levels in first and second trimester of women who proceed to GDM development are demonstrated [75,76]	Significance is lost on multivariate analysis with clinical risk factors [75,76]
Lipids	Elevated triglycerides are seen in women with GDM Reductions in HDL levels are seen in association with GDM [77,78,79,80,81,82,83,84,85]	Predictive and diagnostic capacity is not yet demonstrated [77,78,79,80,81,82,83,84,85]
Inflammatory markers: TNF-α, CRP, IL-6	TNF-α, IL-6 and CRP are elevated in association with GDM [86,87,88,89,90,91,92,93,94,95]	Non-specific Lack of prospective data [86,87,88,89,90,91,92,93,94,95]
Placental GLUT	Altered in response to maternal hyperglycaemia [96,97,98]	Lacks prospective clinical utility [96,97,98]
Epigenetic markers	Several target sites are identified [99,100,101,102,103,104,105,106]	Further identification of epigenetic sites required Offers promise as tools for diagnosis [99,100,101,102,103,104,105,106]

Abbreviations: SHBG, sex hormone binding globulin; HDL, high density lipoprotein; TNF-α, tumour necrosis factor; CRP, C-reactive protein; IL-6, interleukin-6; GLUT, glucose transporter.
